# Unlocking new horizons in oncology: ivonescimab’s dual-target approach to anti-VEGF/PD-1(L1) therapy

**DOI:** 10.3389/fimmu.2025.1599181

**Published:** 2025-11-17

**Authors:** Fan Shi, Luying Yang, Ye Gao, Yan Hou, Qianxin Lv, Feng Cao, Xi Chen, Jianying Zhang, Le Wang

**Affiliations:** 1 State Key Laboratory of Oral & Maxillofacial Reconstruction and Regeneration, National Clinical Research Center for Oral Diseases, Shaanxi Clinical Research Center for Oral Diseases, Department of Oral and Maxillofacial Surgery, School of Stomatology, The Fourth Military Medical University, Xi’an, Shaanxi, China; 2 Digital Dental Center, the Affiliated Hospital of Shaanxi University of Chinese Medicine, Xianyang, Shaanxi, China; 3 Xi'an Institute of Tissue Engineering and Regenerative Medicine, Xi'an, Shaanxi, China

**Keywords:** bispecific antibodies, ivonescimab, immune checkpoint inhibitors, vascular endothelial growth factor, dual-target

## Abstract

Dual blockade of the PD-1/PD-L1 axis, enabling tumor immune evasion, and the VEGF pathway, driving immunosuppression, represents a promising cancer immunotherapy strategy. Combining immune checkpoint inhibitors (ICIs) with antiangiogenics faces toxicity and cost limitations. Bispecific antibodies (BsAbs) targeting both pathways offer a solution. Preclinical and clinical studies demonstrate that simultaneous inhibition enhances antitumor immunity by reversing T-cell exhaustion, normalizing vasculature, and countering immunosuppression. Ivonescimab, a first-in-class PD-1/VEGF BsAb, exemplifies this approach. Approved in China (NMPA, May 2024) for EGFR-mutant non-squamous NSCLC post-TKI failure and included in national insurance (November 2024), it is under global evaluation in solid tumors. PD-1(L1)/VEGF BsAbs like ivonescimab represent a novel therapeutic strategy with potential for improved efficacy and mitigated toxicity compared to combination therapies. Ongoing trials will define broader applications.

## Introduction

Programmed death-1 (PD-1) is an immune checkpoint receptor and that regulates T cell activity by inhibiting immune responses and promoting self-tolerance. Programmed Death Ligand 1 (PD-L1), the natural receptor for PD-1, predominantly expressed on tumor cells, binds PD-1 to activate downstream signaling pathways and suppresses T cell activation, cytokine secretion, and induces apoptosis; Critically, the PD-1/PD-L1 axis is a key mechanism enabling tumor immune evasion (1). Targeting this axis with immune checkpoint inhibitors (ICIs) revitalizes exhausted T cells in the tumor microenvironment (TME), demonstrating significant clinical efficacy in multiple malignancies with manageable toxicity (2). It is well-established that vascular endothelial growth factor (VEGF) can induce local immunosuppressive effects through diversexb mechanisms; Antiangiogenic agents (e.g., VEGF/VEGFR pathway inhibitors) can remodel the immunosuppressive TME, enhancing ICI efficacy by normalizing tumor vasculature to improve perfusion, blocking VEGF-mediated immunosuppression, and preventing endothelial cell-induced T cell apoptosis (3–5).

Preclinical evidence further indicates that VEGF inhibition may upregulate PD-L1, suggesting bidirectional crosstalk between these pathways in sustaining a pro-tumorigenic microenvironment (6, 7). Despite the promise of ICI-antiangiogenic combinations, high costs and toxicity necessitate strategies to optimize therapeutic outcomes (8). One of the most effective strategies to improve overall efficacy is to develop a bispecific antibody simultaneously targeting PD-1/PD-L1 and VEGF could potentially gain higher target binding specificity and enhanced anti-tumor activity, while also improving safety profiles (9). Several such BsAbs, including ivonescimab, PM8002, and HB0025, are now in clinical development. Notably, ivonescimab received approval from China’s National Medical Products Administration (NMPA) in May 2024 for EGFR-mutated advanced non-squamous NSCLC following tyrosine kinase inhibitor failure and was subsequently included in China’s national medical insurance in November 2024 (10). In addition, Global clinical trials of ivonescimab are ongoing. This review explores recent advances and therapeutic potential of ivonescimab, alongside the development of other PD-1(L1)/VEGF BsAbs in solid tumors.

## Ivonescimab: a novel anti-PD-1/VEGF bispecific monoclonal antibody

### Chemistry and mechanism of ivonescimab

**Figure 1:**
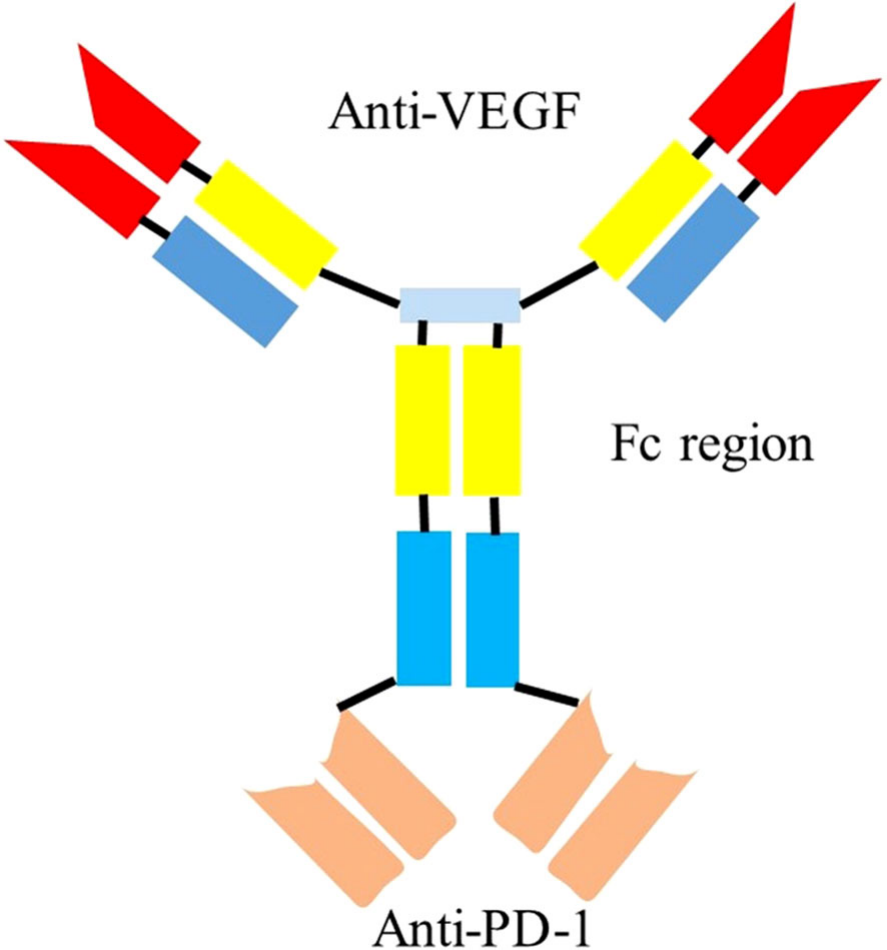
This figure illustrates the tetravalent binding configuration of ivonescimab, an IgG-scFv fusion BsAb developed by Akeso Biopharma. This figure is created with BioRender.com.

**Figure 2:**
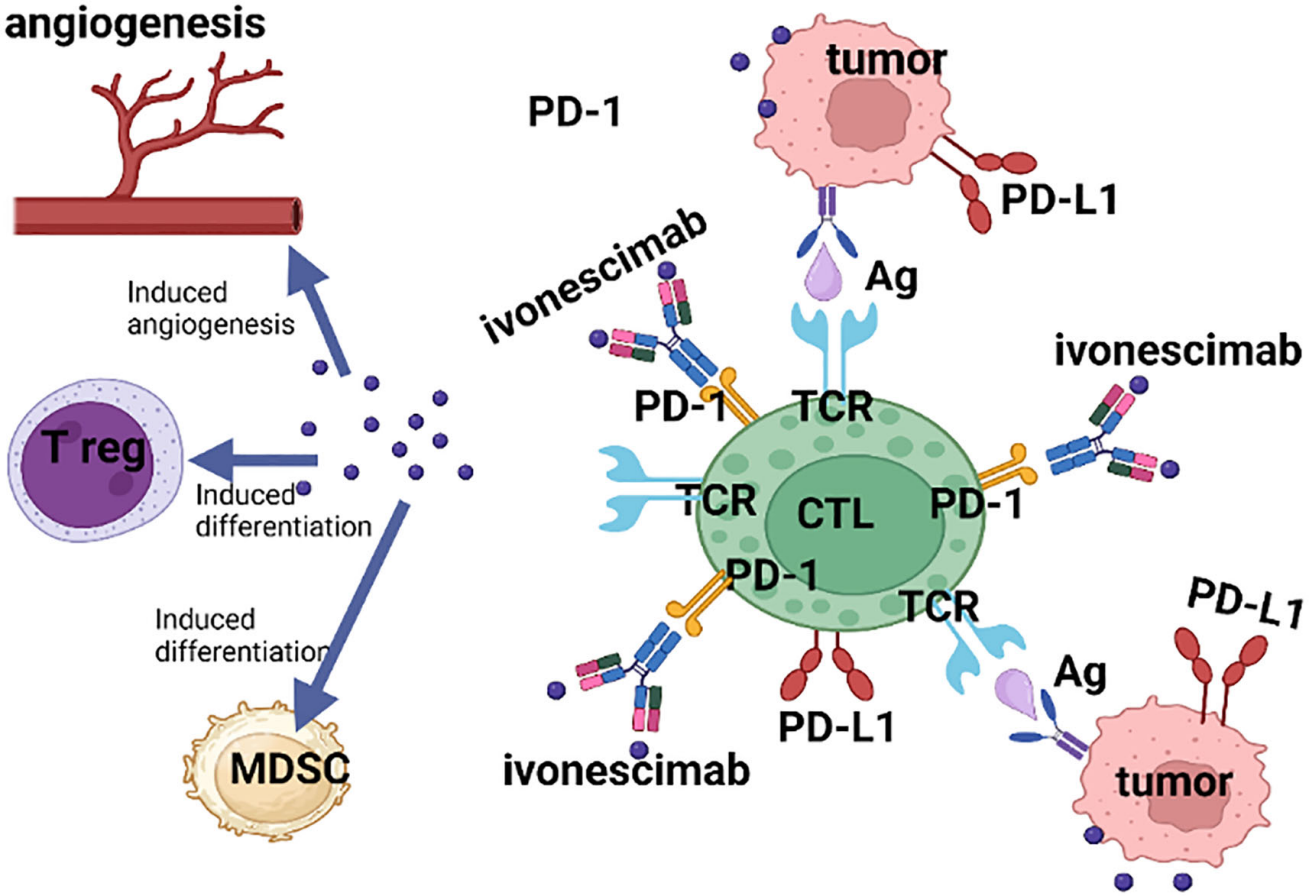
Diagram of the mechanism of action of PD-1/VEGF BsAb (such as Ivonescimab). This figure is created with BioRender.com.

Bispecific antibodies (BsAbs) are a class of engineered proteins that resemble immunoglobulin (IgG) or antibody fragments (Fab), combining various antigen-binding sites into one structure. Their primary goal is to simultaneously block two targets involved in pathological processes, thereby enhancing therapeutic efficacy (11). BsAbs can be created by fusing a single-chain variable fragment (scFv) with the C-terminal Fc fragment of an IgG (i.e., IgG-scFv) (12). Ivonescimab is a pioneering, IgG1-scFv humanized, bispecific monoclonal antibody developed by Akeso Biopharma. It targets both PD-1 and VEGF and is in development for the treatment of non-small cell lung cancer (NSCLC) and other advanced solid tumors, such as breast, liver, and gastric cancer (**Figure 1**). The underlying mechanism of Ivonescimab is as follows (**Figure 2**): 1) In the presence of VEGF, the tetravalent structure of Ivonescimab facilitates the formation of a soluble complex with VEGF dimers. This complex formation accelerates the binding kinetics of Ivonescimab to PD-1, thereby potentiating its blockade of the PD-1/PD-L1 signaling axis and further alleviating PD-1/PD-L1 pathway-mediated immunosuppression, leading to the activation of cytotoxic T lymphocytes (CTLs) and an amplified T-cell response. Furthermore, the antigen-binding activity of Ivonescimab to PD-1 exhibits slower dissociation kinetics in the presence of VEGF, resulting in an 18-fold higher binding affinity compared to penpulimab (11).

2) The binding of ivonescimab to PD-1 reciprocally augments its affinity for VEGF, by effectively sequestering VEGF-A and VEGF-B, ivonescimab inhibits their interaction with VEGFR, and this inhibition disrupts the downstream PI3K/AKT/mTOR signaling cascade, suppressing the proliferation of tumor cells and vascular endothelial cells, and attenuating neovascularization. Concomitantly, this mechanism promotes the infiltration of CTLs into tumor tissues, reprogramming the immunosuppressive tumor microenvironment into an immunologically active state (12).

3) As is well known, the Fc fragment forties the solubility and stability of the protein increases its pharmacokinetic profile, and improves avidity via Fc dimerization, resulting in increased local (near target) antibody concentrations (13). However, for immune checkpoint inhibitors (ICIs), Fc-mediated effector functions can confer detrimental effects. Immune-related adverse events (irAEs) are known to be associated with the recruitment of immune cells expressing Fc receptors. Importantly, the Fc region of ivonescimab has been modified to minimize binding to Fcγ receptors (FcγR), that was engineered to incorporate the double mutation L234A/L235A (termed “DM”), conferring Fc silencing properties. This design eliminates the binding capacity of ivonescimab to FcγRI/IIIa, thereby substantially abrogating Fc-mediated effector functions. These eliminated functions include antibody-dependent cellular cytotoxicity, complement-dependent cytotoxicity, antibody-dependent cellular phagocytosis, antibody-dependent cytokine release, and cytokine release syndrome. This attenuation of Fc functionality may contribute to a reduced risk of irAEs (11).

VEGF and PD-1 are typically co-expressed within the tumor microenvironment. As a PD-1/VEGF BsAb, Ivonescimab not only maintains the activity of the PD-1 monoclonal antibody but also functions as an immune checkpoint inhibitor, further eliminating tumors through effector T cells. Moreover, the combination with VEGF can suppress and block the multiple immunosuppressive effects of VEGF and induce various vascular-modulating responses that enhance immunity. This includes normalizing blood vessels to increase intratumoral blood perfusion and flow, as well as restricting VEGF to promote the differentiation of bone marrow-derived inhibitory T cells. In essence, the dual-target combination increases Ivonescimab’s retention in the tumor microenvironment, further enhancing its anti-tumor activity and achieving an effect where 1 + 1 > 2.VEGF: vascular endothelial growth factor; PD-1:programmed death-1; PD-L1:programmed death ligand 1; Treg: T regulatory cell; MDSC: myeloid-derived suppressor cell; CTL: Cytotoxic T lymphocytes; TCR: T cell receptor; Ag: antigen.

### Preclinical studies of ivonescimab

The results of preliminary research demonstrated that Ivonescimab exhibits high affinity and specific binding to human PD-1 and VEGF; Notably, through SEC-HPLC analysis, it has been detected that Ivonescimab forms soluble complexes with VEGF; Moreover, VEGF efficiently strengthened the binding of Ivonescimab to PD-1, which led to enhanced PD-1 internalization and better potency on blockade of PD-1/PD-L1 signaling. Intriguingly, ivonescimab significantly inhibits tumor growth in a dose-dependent manner in the HCC827 xenograft mouse model (12).

### Pharmacokinetics

Pharmacokinetic (PK) data for ivonescimab were derived from, a multicenter, open-label, dose-escalation phase I study, conducted across five hospitals in China, evaluated ivonescimab monotherapy, with doses ranging from 0.3 to 30 mg/kg administered every two or three weeks (10, 14). After a single intravenous dose, the serum levels of ivonescimab showed a dose-proportional increase within the 3–30 mg/kg range, with steady-state concentrations achieved by the 15th week following repeated administrations. In addition, following single-dose and multiple-dose intravenous administrations of ivonescimab, serum concentration of ivonescimab increased in a dose-dependent manner (9). The maximum concentration (T_max_), mean half‐life (t_1/2_), and mean clearance (CL) did not show obvious dose dependence, and The average accumulation ratio results of Cmax and AUC after multiple dosing indicated that there was a slight accumulation of ivonescimab exposure at steady state (14). Both the initial and fifth doses demonstrated a dose-dependent rise in Cmax for the 0.3, 1, 3, 10, 20, and 30 mg/kg groups dosed biweekly (9). Over time, the clearance rate of ivonescimab declined, starting at 0.461 L/day and stabilizing at 0.334 L/day, marking a 26% reduction. The drug’s elimination half-life was calculated at 9.85 days (10, 14). A population PK analysis indicated that factors such as body weight (ranging from 31 to 155 kg), age, and sex did not significantly influence the drug’s pharmacokinetics. Additionally, total bilirubin levels within the normal range and elevated AST levels did not have a clinically meaningful impact on the drug’s PK profile (14).

### Pharmacodynamics

The pharmacodynamic biomarkers evaluated included PD-1 receptor occupancy (RO) on peripheral CD3+ T cells and serum-free VEGF levels. Following repeated administrations of ivonescimab, high levels of RO were maintained across all dose groups (0.3, 1.0, 3.0, 10.0, 20.0, and 30.0 mg/kg every two weeks), with saturation (>80%) achieved at doses of 3.0 mg/kg and above. Serum-free VEGF concentrations dropped significantly by 80%–95% within 24 hours after the initial dose in all cohorts (9). More importantly, following the first ivonescimab dose, serum VEGF levels decreased relative to baseline in all cohorts except 10 mg/kg Q3W. A consistent pattern of postdose decline followed by gradual recovery was observed after each administration. These VEGF fluctuations showed no clear dose-response relationship. In contrast, the 10 mg/kg Q3W cohort exhibited increased VEGF levels from baseline after the initial dose (14).

## Therapeutic efficacy of Ivonescimab in the clinical study

### Advanced solid tumors

A multicenter, phase I, open-label dose escalation and expansion study (NCT04047290) (9) was conducted in advanced solid tumors that are resistant/refractory to standard therapies, patients received Ivonescimab (0.3 mg/kg to 30 mg/kg) administered IV every 2 weeks (Q2W) and using accelerated titration followed by 3 + 3 + 3 dose escalation design, and results demonstrated that the 47 participants who had at least one postbaseline assessment, the confirmed objective response rate (ORR) was 25.5% and disease control rate (DCR) was 63.8%. Furthermore, results showed that Ivonescimab achieved good antitumor activity and safety in patients with platinum-resistant/refractory epithelial ovarian cancer (PROC), with an ORR of 29.4% and a DCR of 76.5%. The median follow-up duration was 4.5 months, and the median duration of response (DOR) had not yet been reached. Among the four responders, one patient with endometrial cancer had no prior exposure to ICIs or bevacizumab, one patient with ovarian cancer and another with mesothelioma had previously received ICIs, and one patient with microsatellite stable colorectal cancer had undergone prior bevacizumab treatment. Notably, 68.4% of the patients had undergone three or more lines of prior therapy. PR was achieved in five patients, including three with high-grade serous pathology and two with clear cell pathology, resulting in an ORR of 26.3%. The 20 mg/kg dose group demonstrated a higher ORR compared to the 10 mg/kg group (30.0% vs. 14.3%). Additionally, four patients who had previously received bevacizumab experienced stable disease (SD) lasting over 12 months. Promising efficacy signals were also observed in patients with mismatch repair proficient colorectal cancer, non-small cell lung cancer (NSCLC), and both mismatch repair deficient and proficient endometrial cancer. These findings suggest that ivonescimab is well-tolerated at doses up to 20 mg/kg every two weeks (Q2W), with doses of 3 mg/kg Q2W or higher demonstrating significant antitumor activity, achieving an ORR of 23.5% in patients with advanced solid tumors resistant to or relapsed after standard therapies (9).

### Non-small cell lung cancer

A multi-center, open-label Phase Ib/II study (NCT04900363) (15) enrolled 96 patients diagnosed with stage IIIB/C or IV non-small cell lung cancer (NSCLC) to assess the safety and efficacy of Ivonescimab as a first- or second-line monotherapy in advanced NSCLC patients without prior immunotherapy. Participants were administered Ivonescimab intravenously at doses of 10 mg/kg every three weeks (Q3W), 20 mg/kg every two weeks (Q2W), 20 mg/kg Q3W, or 30 mg/kg Q3W. Among the cohort, 66 patients (68.8%) exhibited PD-L1 positivity (tumor proportion score, TPS ≥1%), and 81 patients (84.4%) were treatment-naïve. The study was structured into two phases: dose selection (Phase Ib) and randomized control (Phase II). Across all participants, the overall response rate (ORR) and disease control rate (DCR) were 39.8% and 86.1%, respectively. ORR stratified by TPS was 14.7% for TPS <1%, 51.4% for TPS ≥1%, and 57.1% for TPS ≥50%. In the subset of 67 PD-L1 positive patients receiving first-line Ivonescimab, ORR varied by dose: 33.3% at 10 mg/kg Q3W, 52.6% at 20 mg/kg Q2W, 60.0% at 20 mg/kg Q3W, and 75.0% at 30 mg/kg Q3W.

A phase II, open-label, multicenter clinical trial (NCT04736823) (16) was conducted to assess the safety and efficacy of AK112 combined with chemotherapy in patients with metastatic non-small cell lung cancer (NSCLC). Participants were divided into three cohorts based on their clinical characteristics. Cohort 1 included treatment-naïve patients with advanced NSCLC lacking EGFR or ALK gene alterations. These patients received Ivonescimab alongside pemetrexed (500 mg/m²) for non-squamous (non-sq) NSCLC or paclitaxel (175 mg/m²) for squamous (sq) NSCLC, combined with carboplatin (AUC 5 mg/mL/min) for four cycles. Maintenance therapy involved continued Ivonescimab with pemetrexed for non-sq NSCLC or Ivonescimab monotherapy for sq NSCLC. Cohort 2 comprised patients with EGFR-sensitive mutations who had progressed after EGFR-TKI therapy. They were treated with pemetrexed, Ivonescimab, and carboplatin for four cycles, followed by pemetrexed plus Ivonescimab as maintenance. Cohort 3 included patients with advanced NSCLC who had failed platinum-based chemotherapy and anti-PD-1/PD-L1 therapies, receiving Ivonescimab combined with docetaxel (75 mg/m²). Two Ivonescimab doses (10 or 20 mg/kg) were tested across cohorts, yielding confirmed ORRs of 53.5%, 68.4%, and 40.0% in cohorts 1, 2, and 3, respectively. Additionally, in cohort 1, the median progression-free survival (m-PFS) was not reached, with a 12-month PFS rate of 59.1%. For cohorts 2 and 3, the m-PFS was 8.5 months (95% CI, 5.5–not estimable [NE]) and 7.5 months (95% CI, 2.3–NE), respectively, with corresponding 12-month PFS rates of 35.5% and 44.5%. In summary, among 135 patients with advanced or metastatic NSCLC treated with Ivonescimab plus chemotherapy, 63 had small cell lung cancer (SCLC) and 72 had non-small cell lung cancer (non-SCLC). SCLC patients achieved an ORR of 75%, a median duration of response (DOR) of 15.4 months, a disease control rate (DCR) of 95%, and 9-month PFS and overall survival (OS) rates of 67% and 93%, respectively. Meanwhile, NSCLC patients demonstrated an ORR of 55%, a DCR of 100%, and 9-month PFS and OS rates of 61% and 81%, respectively, though the DOR was not reported.

A global, multicenter, randomized, double-blind Phase III trial (NCT05184712) (17, 18) was conducted to assess the efficacy of Ivonescimab combined with chemotherapy compared to chemotherapy alone in patients with EGFR-mutated, locally advanced, or metastatic NSCLC who had progressed following third-generation EGFR-TKI therapy. In this study, 322 participants were randomized in a 1:1 ratio to receive either Ivonescimab (20 mg/kg) plus pemetrexed (500 mg/m²) and carboplatin or placebo plus chemotherapy every three weeks for four cycles. Among the participants, 86.3% in the Ivonescimab group and 85.1% in the placebo group had previously received third-generation EGFR-TKI therapy, while 21.7% had brain metastases. Stratification was based on prior third-generation EGFR-TKI treatment (received vs. not received) and the presence of brain metastases. Maintenance therapy involved Ivonescimab with pemetrexed or placebo with pemetrexed. After a median follow-up of 7.89 months, the Ivonescimab plus chemotherapy group showed a significant improvement in m-PFS compared to the chemotherapy-alone group (7.06 months vs. 4.80 months, HR 0.46). The m-PFS (95% CI) was 7.06m in the Ivonescimab arm versus 4.8m in the chemotherapy arm. The ORR was 50.6% in the Ivonescimab group (vs 35.4% in the placebo group). Subgroup analyses revealed that nearly all subgroups treated with Ivonescimab exhibited superior PFS compared to placebo. Specifically, patients who had progressed after third-generation EGFR-TKI therapy (HR 0.48, 95% CI 0.35–0.66), those with brain metastases (HR 0.40, 95% CI 0.22–0.73), and individuals with EGFR 19 deletion mutations (HR 0.48, 95% CI 0.35–0.66) or T790M mutations (HR 0.22, 95% CI 0.09–0.54) showed significant benefits. Additionally, the intracranial response rate (IRR) and complete response (CR) rate for the Ivonescimab-chemotherapy combination were 39% and 25%, respectively, while the IRR and CR rate for Ivonescimab monotherapy were both 14%. The median intracranial progression-free survival reached 19.3 months. Therefore, ivonescimab plus chemotherapy significantly improved PFS in the intention-to-treat population. However, the median overall survival data were not mature; at the data cutoff, 69 patients (21.4%) had died.

The randomized, multicenter Phase III clinical trial (NCT05499390) (19) of Ivonescimab vs pembrolizumab in the first-line treatment of PD-L1-positive locally advanced or metastatic NSCLC with PD-L1 positive was performed, and a total of 398 subjects were enrolled, among them PD-L1 tumor proportion score (TPS) 1-49% accounted for 57.8% and PD-L1 TPS ≥50% accounted for 42.2%, and interim analysis revealed that ivonescimab significantly prolonged m-PFS (11.1 vs. 5.8 months), results demonstrated that the primary clinical endpoint of PFS was achieved; The PFS benefit of ivonescimab was observed across patient subgroups, including those with PD-L1 low expression [PD-L1 TPS 1-49%], PD-L1 high expression (PD-L1 TPS > 50%), squamous and nonsquamous histologies, as well as other high-risk clinical features (12). Additionally, Ivonescimab demonstrated higher response rates than pembrolizumab, with ORR (50% vs 39%) and DCR (90% vs 71%); Patients with hepatic metastases demonstrated the most pronounced improvement in the Ivonescimab group (mPFS: 7.1 vs 2.7 months), indicating that adding VEGF inhibition to PD-1/PD-L1 blockade enhances efficacy in this subgroup, and median DoR was not reached in either group.

### Biliary tract cancer

At the 2024 American Society of Clinical Oncology Annual Meeting, a study evaluating the safety and preliminary antitumor activity of ivonescimab combined with chemotherapy in patients with locally advanced or metastatic biliary tract cancer (BTC) was presented, this ongoing, open-label, multicenter, phase 1b/2 study (NCT052114482) (20) was conducted in 22 patients with advanced biliary tract cancer (BTC) in China, including 12 patients with intrahepatic cholangiocarcinoma, one with extrahepatic cholangiocarcinoma and nine with gall bladder cancer, and aimed to evaluate the safety and efficacy of AK112 combined gemcitabine and cisplatin as first-line therapy. The results demonstrated that the ORR and DCR were 63.6% and 100%, respectively after a median duration of 13.8 months (31 January 2024), with eight patients having stable disease; Furthermore, the ORR of gallbladder cancer patients was 77.8%.In addition, the m-PFS and m-OS were 8.5m and 16.8m, respectively.

### Head and neck squamous cell carcinoma

During the ESMO Congress 2024 (Barcelona, Spain), preliminary safety and efficacy data from an investigator-initiated study led by Prof. Chen Xiaozhong were presented as a poster. The study evaluates the novel dual-immunotherapy combination of ivonescimab plus ligufalimab (anti-PD-L1) in the first-line setting for PD-L1-positive recurrent/metastatic head and neck squamous cell carcinoma (HNSCC), this open-label, multicenter, Phase 2 study (NCT04736823) (21) was conducted to assess the safety and efficacy of ivonescimab in combination with Ligufalimab as first-line treatment for PD-L1 positive recurrent/metastasis HNSCC, that enrolled 30 eligible patients with recurrent/metastatic HNSCC who had PD-L1-positive disease (CPS ≥ 1), encompassing oropharyngeal, hypopharyngeal, laryngeal, and oral cancers. These patients received Ivonescimab (10 mg/kg Q3W) as monotherapy or in combination with Ligufalimab (45 mg/kg Q3W). All patients (100%) had an ECOG performance status score of 1, and among them, 56.7% had a CPS≥20. Efficacy data showed: that in the Ivonescimab monotherapy group, among 10 patients, the ORR was 30.0% the DCR was 80.0%, the median DoR was not reached, and the median PFS was 5.0 months. In addition, in the Ivonescimab combined with Ligufalimab group, among 20 patients, the ORR and DCR were 60.0% and 90.0% respectively, the median DoR was not reached, the median PFS was 7.1 months, and the 6-month PFS rate was 71.8%. Among the 11 patients with CPS ≥ 20 in the Ivosidenib + Lifralimab group, the ORR and DCR were 72.7% and 81.8% respectively, while among the 9 patients with 1 ≤ CPS < 20, the ORR and DCR were 44.4% and 100.0% respectively.

### Small cell lung cancer

A phase Ib trial (22) enrolled 35 treatment-naïve patients with extensive-stage small cell lung cancer (ES-SCLC) to evaluate the safety and efficacy of Ivonescimab in combination with etoposide and carboplatin. Participants received intravenous Ivonescimab every three weeks at doses of 3 mg/kg, 10 mg/kg, or 20 mg/kg, with the treatment regimen lasting up to four cycles, followed by Ivonescimab monotherapy as maintenance. After a median follow-up of 13.3 months, the ORR was 80%, with measurable tumor lesions shrinking by over 30%, and the DCR reached 91.4%. Among the three dose groups, the ORRs were 66.7% for 3 mg/kg, 90.9% for 10 mg/kg, and 76.2% for 20 mg/kg, with the 10 mg/kg dose demonstrating the highest efficacy.

### Triple-negative breast cancer

Building on the favorable outcomes observed with prior combinations of immunotherapy, anti-angiogenic therapy, and chemotherapy in breast cancer, Professor Wang Xiaojia presented preliminary findings from a study investigating first-line ivonescimab plus chemotherapy for advanced triple-negative breast cancer (TNBC) in an oral presentation at the 2024 European Society for Medical Oncology (ESMO) Congress, this the open-label, multicenter, Phase 2 study (NCT05227664) (23) was conducted to assess the safety and efficacy of ivonescimab in combination with chemotherapy as a first-line treatment for TNBC. A total of 30 patients were enrolled, with 80% of them having a PD-L1 combined positive score (CPS) <10. Additionally, 60% of the patients had previously undergone neoadjuvant or adjuvant therapy based on taxoid drugs. The patients were administered ivonescimab at a dosage of 20 mg/kg every two weeks in conjunction with paclitaxel 90 mg/m² or nab-paclitaxel 100 mg/m² on days 1, 8, and 15 of each four-week treatment cycle. The median follow-up duration was 7.2 months, during which 29 patients underwent at least one post-baseline tumor evaluation. The investigator-assessed ORR was 72.4% (21/29), with a DCR of 100% (29/29). Among the patients, those with a PD-L1 CPS of ≥10 had an ORR of 83.3% (5/6), while those with a PD-L1 CPS of <10 had an ORR of 69.6% (16/23). Median PFS and OS data are not yet available, but the 6-month PFS rate was 68.4%.

### Metastatic colorectal cancer

At the 2024 ESMO Congress, specifically within the Gastrointestinal Cancers track, Professor Deng Yanhong, serving as the leading investigator, presented an oral report on the latest findings from an open-label, multicenter, randomized phase II clinical trial (NCT05382442) (24) was conducted to evaluate the efficacy and safety of Ivonescimab in the treatment of metastatic colorectal cancer (mCRC), that untreated mCRC patients were randomly assigned in a 1:1 ratio to two treatment groups: Group A received FOLFOXIRI combined with Ivonescimab, while Group B received the same regimen as Group A with the addition of Ligufalimab. Both groups underwent a maximum of 8 cycles of treatment, followed by maintenance therapy with 5-fluorouracil plus Ivonescimab (Group B) or 5-fluorouracil alone (Group A), with or without Ligufalimab. A total of 40 mCRC patients were enrolled and randomly assigned to Group A (n=22) and Group B (n=18). Notably, 11 patients in both Group A and Group B had KRAS/BRAF mutations. Except for one patient with an unknown status, all other patients were microsatellite stable (MSS). The median follow-up duration was 9 months for Group A and 9.6 months for Group B, of Group A and Group B were 81.8% and 88.2%, respectively. More impressively, the DCR was 100% in both groups. Median PFS and OS data are not yet mature. However, the 9-month PFS rate was 81.4% (95% CI:52.1%-93.7%) for Group A and 86.2%(95% CI:55%-96.4%)for Group B.

## The safety and tolerability of ivonescimab

Ivonescimab demonstrated a higher incidence of treatment-related adverse events (TRAEs) versus pembrolizumab or control groups. TRAEs with ivonescimab were consistent with known toxicities of PD-L1 and VEGF inhibitors (19). The predominant TRAEs included proteinuria, elevated aspartate aminotransferase, hypercholesterolemia, hyperbilirubinemia, neutropenia, leukopenia, thrombocytopenia), anemia, and hypertension. The incidence of grade ≥3 TRAEs was higher with ivonescimab versus pembrolizumab. Serious AEs occurred more frequently in the ivonescimab group, with disease progression, thrombocytopenia, anemia, pneumonitis, COVID-19 infection, and hepatic dysfunction being the most prevalent. Treatment-emergent AEs leading to death were reported in 10.6% of ivonescimab-treated patients (all attributed to disease progression), versus 7.5% (12 cases) in the placebo group (11/12 due to disease progression). Consequently, SAEs were more common with ivonescimab than with placebo or pembrolizumab. Immune-related adverse events (irAEs) occurred at higher rates with ivonescimab (24.2% vs 6.2% placebo). The most frequent grade ≥3 irAEs were rash, dermatitis, interstitial lung disease, pneumonitis, hepatic dysfunction, and hypothyroidism. VEGF inhibition-related adverse events predominated in the ivonescimab group, notably grade ≥3 hypertension (5% vs 1%) and proteinuria (3% vs 0%). Importantly, grade ≥3 hemorrhage rates were comparable (1% vs 1%). Notably, grade ≥3 irAEs and overall grade ≥3 AEs occurred at similar frequencies between ivonescimab and placebo. Safety profiles were consistent across histologic subtypes (squamous vs non-squamous NSCLC) and comparable to pembrolizumab in squamous NSCLC. Overall, ivonescimab’s safety profile appears manageable, particularly given its substantial progression-free survival benefit (18, 19, 25).

### The advantages and disadvantages of PD-1(L1)/VEGF BsAbs

These PD-1(L1)/VEGF BsAbs aim to block two protumor signaling pathways, potentially producing synergistic anti-cancer effects or minimizing drug resistance, and represent a novel class of drugs that have made significant advancements in recent years. Hence, we summarize the structure characteristic and clinical maturity (**Table 1**), and the status and efficacy of several PD-1(L1)/VEGF BsAbs entering clinical trials (**Table 2**, **Supplementary Table 1**). In addition, PD-1(L1)/VEGF BsAbs will provide several superiority over other anti-PD-1(L1) and anti-VEGF antibodies drug as follows: 1) Enhanced efficacy: Compared with ordinary monoclonal antibodies, bispecial antibodies can bind two specific epitopes or target proteins simultaneously, so they can play a special function to play a biological function that is difficult to achieve with monoclonal antibodies; 2) BsAbs containing Fc region: Have good solubility and stability, a long half-life, play antibody-dependent cell-mediated cytotoxicity effect to further enhance the tumor killing effect; 3) Prevention of drug resistance: simultaneously blocking two different signal pathways with unrelated or overlapping pathogenic functions; 4) Low toxicity: Compared with monoclonal antibody, BsAbs have stronger specificity and targeting, reducing off-target toxicity; 5) Low cost: Compared with the combination therapy of monoclonal antibody, it can also effectively reduce the cost of treatment (5, 16). As is well known, the inhibition of the VEGF–VEGFR axis has numerous favorable effects that can, in principle, enhance the efficacy of immunotherapy. However, PD-(L)1/VEGF BsAbs exhibit certain limitations, as exemplified by the clinically approved agent ivonescimab: 1) in subgroup analyses, patients with TPS ≥50% consistently demonstrated superior response rates compared to those with TPS 1%-49%, this observation aligns with the dual targeting mechanism of ivonescimab against both PD-1 and VEGF. Notably, squamous NSCLC patients exhibited a 13% higher ORR than non-squamous counterparts, whereas non-squamous NSCLC showed prolonged m-PFS. These discordant outcomes underscore the complexity of treatment responses across histological subtypes and warrant further investigation to elucidate underlying mechanisms; 2) The exclusive reporting of PFS without corresponding OS data precludes comprehensive assessment of therapeutic benefit, particularly given the documented dissociation between PFS and OS in the immunotherapy era. 3) the study’s confinement to a single Chinese cohort raises concerns regarding generalizability across ethnic and geographic populations. Consequently, while demonstrating therapeutic promise, ivonescimab’s global applicability requires validation through multinational trials—specifically examining OS in diverse populations.

**Table 1:** Comparison of Ivonescimab with other anti-VEGF/PD-1(L1) BsAb in molecular architecture and clinical maturity.

Comparison dimension	Ivonescimab	PM8002	IMM2510	HB0025
molecular architecture	Heterotetramer structure, anti-PD-1 ScFv is attached to the C-terminal of each anti-VEGF antibody heavy chain	anti-PD-L1 single-domain antibody was fused onto an anti-VEGF-A IgG1 antibody containing the FC-silencing mutation	mAb-Trap structure, It is composed by the fusion of anti-PD-L1 antibodies with VEGFR	second Ig-like domain outside the VEGFR1 membrane is connected to the N-terminal of the IGG1-type anti-PD-L1 mAb heavy chain through a flexible linker
FC region	mutation sites (L234A and L235A) were introduced into the fc region. leads to a silent mutation in Fc, block the binding of FcγRI/IIIA	FcγRIIB binding enhanced mutation	No Fc silencing mutation exerts the ADCC effect by binding to the Fcγ receptor	No Fc silencing mutation
Clinical Maturity	NSCLC Indication Approval (China) Three Phase III projects completed Data of over 2,000 patients	Phase Ib/II is in progress Solid tumor • Public data: n=87	Phase I dose expansion • Advanced solid tumor Only disclose the security of n=24	Phase I completed Solid tumor • Publish n=24

**Table 2:** Comparison of Ivonescimab with other anti-VEGF/PD-1(L1) BsAb in clinical trials.

Drug	Phase	Tumor type	subclassification	ORR (%)	DCR (%)	m-PFS (months)	m-DOR (months)	AEs (%)	≥grade 3 AEs(%)	Reference
Ivonescimab	Ia	Solid tumor		25.5	63.8	–	–	74.5	27.5	(9)
	Ia	PROC		26.3	76.5	13.5	NR	88.5	15.8	(9)
	II	NSCLC		39.8	86.1	–		91.6	51.8	(16)
	Ib	NSCLC	TPS<1%	14.7	–	–	–	–		(15)
			1%≤TPS<50%	51.4	–	–	–	–		
			TPS>50%	57.1	–	–	–	–		
	II	NSCLC	sq-NSCLC	75	95	–	15.4	–	–	(16)
			Non-sq-NSCLC	55	100	–	NR			
	III	NSCLC EGFR Variant		50.6		7.1	–	–	61.5	(18)
Ivonescimab	II	BTC		63.6	100	8.5	NR	–	86.4	(16)
Ivonescimab	II	SCLC		80	91.4	–	–	–		
Ivonescimab	II	TNBC		72.4	100	9.3	7.49	–	-	
Ivonescimab	II	HNSCC	Ivonescimab	30	80	5	–	–	–	
			Ivonescimab+ligufalinab	60	90	7	–	–	–	
Ivonescimab	II	mCRC	Ivonescimab+FOLFOXIRI	81.8	100	–				
			Ivonescimab+FOLFOXIRI+ ligufalinab	88.2	100	–				
HB0025	I	Solid tumor		9.1	50	–	–	83.3	20	(26)
IMM2510	I	Solid tumor		75.8	–	–	–	97	27.4	(27)
PM8002	I	Solid tumor	dose escalation	19.7	72.7	–	–	95.4	35.6	(28)
			dose expansion	20	70	–	–			
	Ib/II	NSCLC		79	77	–	–	85.2	18	(29)
	II	SCLC		72.7	81.8	5.5	–	93.8	62.5	(30)
	Ib/II	TNBC		78.6	95.2	9.2	7.2	95.2	38.1	(31)

ORR, the objective response rate; DCR, disease control rate; PR, partial responses; SD, stable disease; PD, progressive disease; SCLC, small cell lung cancer; sq-NSCLC, squamous non-small cell lung cancer; Nonsq-NSCLC, nonsquamous non-small cell lung cancer; DOR, duration of response; TRAEs, treatment-related adverse events; ICI, immune checkpoint inhibitor; NK, ADCC/ADCP; TNBC, triple-negative breast cancer; CC, cervical cancer; PROC, Platinum-resistant ovarian cancer; NR, not reached. Cohort 1, patients receiving first-line Ivonescimab in combination with platinum-based chemotherapy; Cohort 2, patients with EGFR-sensitive mutations who failed previous targeted therapy; Cohort 3, patients who failed previous systemic platinum-based chemotherapy and PD-1/L1 inhibitor treatments; HNSCC, Head neck squamous cell Carcinoma; mCRC, metastatic colorectal cancer.

Ivonescimab reactivates dysfunctional T cells through PD-1 blockade, concurrently remodels the tumor vasculature, and reverses immunosuppressive signaling via VEGF inhibition. However, it is worth noting that althoughIvonescimab can induce long-lasting responses in some refractory tumors, they paradoxically trigger elevated PD-L1 expression within the tumors and increase the population of regulatory T cells (Tregs) in the TME, thereby attenuating their antitumor efficacy (28). Furthermore, it is widely accepted that non-exhausted T cells within the tumor microenvironment constitute the key effector mechanism of immunotherapy, while T cell exhaustion and senescence represent critical pathways enabling cancer cells to evade immune surveillance, sustain an immunosuppressive microenvironment, and drive resistance to cellular immunotherapies (28).

The core mechanism of action of ivonescimab involves blockade of key inhibitory signaling pathways, primarily the PD-1/PD-L1 axis, and this blockade aims to reinvigorate exhausted T cells and restore their anti-tumor functionality. However, prolonged exposure to the antibody may paradoxically promote the progression of T cells into a state of deeper exhaustion (29). For instance, under conditions of persistent antigen exposure, a sustained immunosuppressive TME, and adaptive tumor evolution, T cells may compensate by upregulating alternative inhibitory receptors. This compensatory upregulation can maintain and drive T cells towards a terminally exhausted phenotype, while simultaneously facilitating the development of novel immune evasion mechanisms, ultimately leading to acquired resistance (30). Furthermore, following successful T cell activation by ivonescimab, the continued presence of unresolved tumor antigens subjects these T cells to chronic activation. Such sustained, high-intensity stimulation is intrinsically a critical driver of T cell exhaustion. Additionally, profoundly exhausted T cells undergo stable epigenetic reprogramming, which functionally locks them into an irreversible dysfunctional state (32).

In addition, from our perspective, some potential issues also deserve attention if the antiangiogenic function of drugs reduces tumor vascularity and thus overall tumor blood vessel perfusion and flow, one might expect at least two unfavorable functional outcomes that, alone or together, could impair the immunotherapeutic properties of the drug: (1) increased levels of tumor hypoxia — a known mediator of resistance to immunotherapy; and (2) impaired intratumoral antibody biodistribution, thus reducing immunoefficacy (5, 33–35). Therefore, the final clinical benefits of anti-VEGF/PD-1(L1) BsAb will depend on the net balance between the opposing effects of VEGF signaling and its inhibition on the antitumor immune response in a given treatment situation.

### Ongoing clinical trials

Ivonescimab has been approved in combination with pemigatinib and carboplatin for the treatment of patients with advanced or metastatic NSCLC who have progressed after receiving treatment with an EGFR tyrosine kinase inhibitor and have tested positive for EGFR gene mutation. In addition, there are 5 Phase III clinical studies underway (**Table 3**), including 2 international multi-center Phase III clinical studies conducted overseas, and 4 Phase III clinical studies using PD-1 monoclonal antibodies as positive control drugs.

**Table 3:** Ongoing clinical trials of Ivonescimab.

Clinical registration number	Disease	Regimen	Phase	Single/multicenter	Number of participants
NCT06396065	nsq-NSCLC	ivonescimab+ Pemetrexed _+_ carboplatin	III	International multicenter	420
NCT05899608	sq-NSCLC	ivonescimab vs Keytruda + chemotherapy	III	International multicenter	400
NCT05840016	sq-NSCLC	ivonescimab vs tislelizumab + chemotherapy	III	Single center	396
NCT05499390	NSCLC	ivonescimab vs Keytruda	III	Single center	388
NCT05184712	nsq-NSCLC	ivonescimab	III	International multicenter	470
NCT04870177	gynae- ecological tumours	ivonescimab	II	multicenter	270
NCT05229497	malignant tumors	ivonescimab +ligufalimab	Ib/II	multicenter	114
NCT05432492	HC	ivonescimab	II	multicenter	–
NCT05247684	NSCLC	ivonescimab	II	multicenter	–

## Further perspective and challenges

The development of BsAbs, including anti-VEGF/PD-1(L1) and similar constructs, represents a focused effort to improve anti-tumor immune responses, counteract immune evasion mechanisms, and address the limitations of single-agent therapies. Although early clinical successes highlight the potential of BsAbs, their effective incorporation into cancer treatment regimens demands careful planning.

Key areas for future investigation include enhancing the precision, effectiveness, and safety profiles of BsAbs. This involves optimize the design of BsAb with lower immunogenicity, improved tumor-targeting capabilities, and optimized pharmacokinetic properties, as well as ensuring selective accumulation in the TME to reduce systemic toxicity. Additionally, identifying predictive biomarkers for BsAb therapy is critical. Comprehensive analyses of tumor genomics, proteomics, and immunology may uncover biomarkers that guide personalized treatment and reduce adverse effects; furthermore, comprehensive multi-omics analyses are critical to identify predictive biomarkers for patient stratification, mitigating off-target effects and maximizing therapeutic efficacy. Combining BsAbs with other therapies, such as chemotherapy, targeted agents, radiation, and additional immunotherapies, also shows significant potential. Evidence-based combination strategies can amplify anti-tumor effects and expand the therapeutic scope of BsAbs. However, rational combination strategies with other therapies require mechanistic evidence to amplify anti-tumor responses without compounding toxicity; cost-effectiveness analyses must further guide clinical adoption. Crucially, the TME plays a pivotal role in the efficacy of BsAbs in solid tumors. Thus, advancing BsAbs that modulate suppressive TME components, enhance antigen presentation, and promote T cell infiltration and activation is essential. Given the TME’s pivotal role, next-generation BsAbs should actively remodel immunosuppressive elements (e.g., Tregs, MDSCs), enhance antigen presentation, and promote T-cell infiltration/activation. Spatial heterogeneity of the TME warrants dedicated investigation. Finally, further research into the PK and PD of BsAbs, *(*particularly organ-specific toxicities like hepatotoxicity or cytokine release syndrome*)*, and real-world evidence generation are essential to refine dosing and safety monitoring, along with elucidating their mechanisms of action and potential organ toxicity, is necessary to improve targeting accuracy and minimize off-target effects.

## Conclusion

The China National Medical Products Administration approved ivonescimab, in combination with pemetrexed and carboplatin for the treatment of patients with EGFR-mutated locally advanced or metastatic non-squamous NSCLC who have progressed after tyrosine kinase inhibitor therapy on May 23, 2024. This therapy targets two distinct antigen-binding sites involved in immune regulation, representing a pivotal evolution in the treatment landscape for advanced or metastatic NSCLC patients who have progressed after receiving treatment with an epidermal growth factor receptor tyrosine kinase inhibitor and have EGFR gene mutation positivity. Looking ahead, it is highly likely that more anti-VEGF/PD-1(PD-L1) BsAbs will also demonstrate potential for approval in treating other cancers, such as TNBC and SCLC.

## Supplementary material

Supplementary Table 1 Comparison of Ivonescimab with other anti-VEGF/PD-1(L1) BsAb in clinical trials.
