# Supplementary material for: Unlocking new horizons in oncology: ivonescimab’s dual-target approach to anti-VEGF/PD-1(L1) therapy
Source: Front Immunol. 2025 Nov 17;16:1599181. doi: 10.3389/fimmu.2025.1599181 (PMC12665695; doi:10.3389/fimmu.2025.1599181)
Supplement: Supplementary Table 1 — Comparison of Ivonescimab with other anti-VEGF/PD-1(L1) BsAb in clinical trials. [file Table1.docx]

Table 1 Comparison of Ivonescimab with other anti-VEGF/PD-1(L1) BsAb in clinical trials

| Drug | phase | Tumor type | subclassification | ORR  (%) | DCR  (%) | m-PFS  (months) | m-DOR  (months) | AEs  (%) | ≥grade 3 AEs(%) | Reference |
| --- | --- | --- | --- | --- | --- | --- | --- | --- | --- | --- |
| Ivonescimab | Ia | Solid tumor |  | 25.5 | 63.8 | - | - | 74.5 | 27.5 | ^[9]^ |
|  | Ia | PROC |  | 26.3 | 76.5 | 13.5 | NR | 88.5 | 15.8 | ^[9]^ |
|  | II | NSCLC |  | 39.8 | 86.1 | - |  | 91.6 | 51.8 | ^[18]^ |
|  |  | Cohort 1 |  | 53.5 | 93 | NR | NR | - | - |  |
|  |  |  | non-sq-NSCLC | 48.0 | - | - | - | - | - |  |
|  |  |  | sq-NSCLC | 61.1 | - | - | - | - | - |  |
|  |  | Cohort 2 |  | 68.4 | 94.7 | 8.5 | 8.38 | - | - |  |
|  |  | Cohort 3 |  | 40.0 | 70 | 7.5 | NR | - | - |  |
|  | Ib | NSCLC | TPS＜1% | 14.7 | - | - | - | - |  | ^[17]^ |
|  |  |  | 1%≤TPS＜50% | 51.4 | - | - | - | - |  |  |
|  |  |  | TPS＞50% | 57.1 | - | - | - | - |  |  |
|  | II | NSCLC | sq-NSCLC | 75 | 95 | - | 15.4 | - | - | ^[18]^ |
|  |  |  | Non-sq-NSCLC | 55 | 100 | - | NR |  |  |  |
|  |  | EGFR/ALK wild type | Non-sq-NSCLC | 54.2 | 95.8 | NR | 15.4 | - | - |  |
|  |  |  | Sq-NSCLC | 71.4 | 90.5 | 11.1 | 12.7 | - | - |  |
|  |  | EGFR-TKI treatment failed for EGFR mutations | Sq-NSCLC | 68.4 | 94.7 | 8.5 | 8.7 | - | - |  |
|  |  | PD1/PDL1 mAb +platinum chemotherapy failed | NSCLC | 40 | 80 | 7.5 | 12.7 | - | - |  |
|  | III | NSCLC  EGFR Variant |  | 50.6 |  | 7.1 | - | - | 61.5 | [21] |
| Ivonescimab | II | BTC |  | 63.6 | 100 | 8.5 | NR | - | 86.4 | [22] |
| Ivonescimab | II | SCLC |  | 80 | 91.4 | - | - | - |  |  |
|  |  |  | Doagse 30mg/kg | 66.7 | - | - | - | - | 66.7 |  |
|  |  |  | Doagse 10mg/kg | 90.9 | - | - | - | - | 54.5 |  |
|  |  |  | Doagse 20mg/kg | 76.2 | - | - | - | - | 61.9 |  |
| Ivonescimab | II | TNBC |  | 72.4 | 100 | 9.3 | 7.49 | - | - |  |
|  |  |  | PD-L1 CPS≥10 | 83.3 | - | NR | NR | - | - |  |
|  |  |  | PD-L1 CPS＜10 | 69.6 | - | 9.3 | 7.49 | - | - |  |
| Ivonescimab | II | HNSCC | Ivonescimab | 30 | 80 | 5 | - | - | - |  |
|  |  |  | Ivonescimab+ligufalinab | 60 | 90 | 7 | - | - | - |  |
|  |  |  | CPS≥20 | 72.7 | 81.8 |  |  |  |  |  |
|  |  |  | 1≤CPS＜20 | 44.4 | 100 |  |  |  |  |  |
| Ivonescimab | II | mCRC | Ivonescimab+FOLFOXIRI | 81.8 | 100 | - |  |  |  |  |
|  |  |  | Ivonescimab+FOLFOXIRI+ ligufalinab | 88.2 | 100 | - |  |  |  |  |
| HB0025 | I | Solid tumor |  | 9.1 | 50 | - | - | 83.3 | 20 | ^[30]^ |
| IMM2510 | I | Solid tumor |  | 75.8 | - | - | - | 97 | 27.4 | ^[31]^ |
| PM8002 | I | Solid tumor | dose escalation | 19.7 | 72.7 | - | - | 95.4 | 35.6 | ^[32]^ |
|  |  |  | dose expansion | 20 | 70 | - | - |  |  |  |
|  |  | CC |  | 42.2 | 93.3 | 8.3 | - | - | - |  |
|  |  | PROC |  | 20.6 | 67.7 | 5.3 | - | - | - |  |
|  | Ib/II | NSCLC |  | 79 | 77 | - | - | 85.2 | 18 | ^[33]^ |
|  | II | SCLC |  | 72.7 | 81.8 | 5.5 | - | 93.8 | 62.5 | ^[34]^ |
|  |  |  | Did not receive immunotherapy | 72.7 | - | 5.9 | - | - | - |  |
|  |  |  | receive immunotherapy | 42.9 | - | 3.9 | - | - | - |  |
|  | Ib/II | TNBC |  | 78.6 | 95.2 | 9.2 | 7.2 | 95.2 | 38.1 | ^[35]^ |

Abbreviation: ORR: the objective response rate; DCR: disease control rate; PR: partial responses; SD: stable disease; PD: progressive disease; SCLC: small cell lung cancer; sq-NSCLC: squamous non-small cell lung cancer; Nonsq-NSCLC: nonsquamous non-small cell lung cancer; DOR: duration of response; TRAEs: treatment-related adverse events; ICI: immune checkpoint inhibitor; NK: ADCC/ADCP;TNBC: triple-negative breast cancer; CC: cervical cancer; PROC: Platinum-resistant ovarian cancer; NR: not reached. Cohort 1: patients receiving first-line Ivonescimab in combination with platinum-based chemotherapy; cohort 2: patients with EGFR-sensitive mutations who failed previous targeted therapy;  cohort 3: patients who failed previous systemic platinum-based chemotherapy and PD-1/L1 inhibitor treatments; HNSCC: Head neck squamous cell Carcinoma; mCRC: metastatic colorectal cancer.
